# District Nursing: I. A Historical Sketch

**Published:** 1918-06-08

**Authors:** 


### THE MATRONS' AND SISTERS' DEPARTMENT.

### DISTRICT NURSING.

### I.—A Historical Sketch.

The St. Katherine's Royal Hospital, latterly moved to Regent's Park from its old home near the Tower of London, dates from the reign of King Stephen, whose queen, Matilda, founded it. It was a religious foundation, and always remained under the patronage of the Queens of England. One of the principal duties of the original sisters of the community was to visit and care for the sick-poor in the neighbourhood. Owing probably to its royal protection and the lowly sphere of its activities, St. Katherine's escaped destruction at the Reformation, and in 1878 Queen Victoria revived its old activities by appointing several nurses to attend cases of sick-poor in whom she was personally interested. These nurses received a grant from St. Katherine's, wore the royal monogram as a brassard, and were called Queen's nurses. A few years later they were absorbed by the newly incorporated Queen Victoria's Jubilee Institute, which was at first, by the Queen's own desire, connected with the old foundation, housed in its precincts, and presided over by its master. This intimate connection proved very temporary, but some satisfaction of the historic sense is served by this linking-up of the great modern, highly specialised, intensely practical organisation with those shadowy sisters of the past who "went about doing good" among the sick-poor around them.

The birth of modern nursing in England was Elizabeth Fry's "Training Institute for Nurses," founded in 1840. The nurses got some teaching and then went to work in hospitals and private families. Apparently from the first they worked between their cases for the sick-poor around them. A few years later there is record of their being engaged by clergymen for parish nursing. Several similar institutes were founded in the next few years.

The real birthplace of district nursing was Liverpool, and its founder was Sir William Rathbone, M.P. Lady Rathbone had experienced much relief and comfort from the attendance of a trained nurse during her last illness. After her death, in 1859, Sir William Rathbone made the experiment of engaging the nurse who had nursed his wife and placing her to work among the very poor of a district in Liverpool. The result of the experiment was so satisfactory that Sir William wanted immediately to place more nurses at the same work, but the London training-schools had no nurses to send. So Liverpool, with the help of Florence Nightingale, started a hospital training-school of its own, and in a few years Liverpool had eighteen district nurses and a superintendent. This was the first District Nursing Association.

London shortly followed suit with the East London Nursing Society and the Nursing Branch of the London Biblewomen, and in 1874 the English branch of the Order of St. John of Jerusalem was instrumental in founding "The National Association of Trained Nurses for the Sick-Poor. The object of the Association was the organisation of district nursing, first in London, and then throughout the country. It made very useful criticism of the existing work, which tended to consist of too much almsgiving and too little nursing, and it founded the "Central Home and Training-School" in Bloomsbury Square, with Miss Florence Lees as superintendent. The Association was very strongly convinced of the great value of the work, and of the desirability of keeping it in the hands of trained women of the educated classes. The nurses had full hospital training (it was a year in those days), followed by six months' special district training at the Bloomsbury Home. The first nurses met with some criticism; their unchaperoned walks abroad shocked some people, their empty hands disappointed others. It was a definite rule from the first that where material help was necessary it should not come through the nurse. However, the new nurses were soon in great demand, and as soon as sufficient staff could be trained various other London centres were founded.

In 1887 Queen Victoria decided to devote the Women's Jubilee Offering to the development of district nursing. It was made the original endowment of the "Queen Victoria's Jubilee Institute," which was for the future to be the national organisation for the whole of Great Britain. The new institute grew rapidly; it absorbed and affiliated Bloomsbury and the other existing centres, and steadily developed the work till from its headquarters in Victoria Street it controlled and standardised the work of several thousand women. The original endowment has been increased from time to time to enable it to cope with the work. Locally the institute has always advocated a provident basis wherever possible.

Excellent district nursing is also done outside the Q.V.J.I. Various religious bodies provide nurses for their poor. Some local associations have never affiliated. So something remains to desire. The work has been steadily developing for over thirty years, and is now universally recognised as having not only a humane, but an educational value. Charles Booth described it as "the most directly satisfactory" of all forms of charity, and continued that "it is almost true to say that wherever a district nurse enters, there the standard of life is raised."

